# Non-calcified plaque volume assessed by coronary computed tomographic angiography: prognostic significance, reproducibility, and temporal progression

**DOI:** 10.1093/ehjimp/qyag115

**Published:** 2026-08-27

**Authors:** Salman Ansari, Matthew J Budoff

**Affiliations:** Department of Internal Medicine, Riverside Community Hospital, 4445 Magnolia Ave, Riverside, CA 92501, USA; Department of Medicine, Lundquist Institute at Harbor-UCLA Medical Center, Los Angeles, CA, USA

**Keywords:** non-calcified plaque volume, coronary CT angiography, atherosclerosis, ICONIC, SCOT-HEART, PARADIGM

## Abstract

**Aims:**

Coronary artery disease remains the leading cause of cardiovascular mortality worldwide, with a substantial proportion of acute coronary events arising from rupture of non-calcified, lipid-rich plaques not detected by conventional coronary artery calcium scoring. Coronary computed tomographic angiography (CCTA) enables direct visualization and volumetric quantification of non-calcified plaque volume (NCPV), capturing a biologically active component of coronary atherosclerosis. No comprehensive synthesis of NCPV's prognostic significance, measurement reproducibility, and pharmacological modifiability has been published. This review addresses that gap.

**Methods and results:**

We conducted a narrative review of published literature through March 2025 using PubMed, synthesizing evidence from multicentre trials and registry analyses. Studies including SCOT-HEART, ICONIC, CONFIRM, and PARADIGM demonstrate that NCPV is associated with major adverse cardiovascular events independent of coronary artery calcium score, cardiovascular risk scores, and stenosis severity. Low-attenuation plaque burden represents one of the strongest predictors of myocardial infarction. Non-obstructive high-risk plaques confer risk comparable to obstructive lesions without high-risk features. Sex-related differences in plaque composition are clinically relevant, with women demonstrating lower fibrous and fibrofatty plaque volumes than men. Artificial intelligence-based quantitative CCTA has improved reproducibility, with interobserver variability below 3%. Serial imaging demonstrates that NCPV is modifiable with intensive lipid-lowering therapy.

**Conclusion:**

NCPV assessed by CCTA represents a reproducible and prognostically relevant measure of coronary atherosclerosis that is responsive to therapy. Recent regulatory interest, including submission of a Biomarker Qualification Program Letter of Intent to the US Food and Drug Administration, highlights its potential role as a biomarker for clinical trial enrichment.

## Graphical Abstract

**Graphical Abstract:**
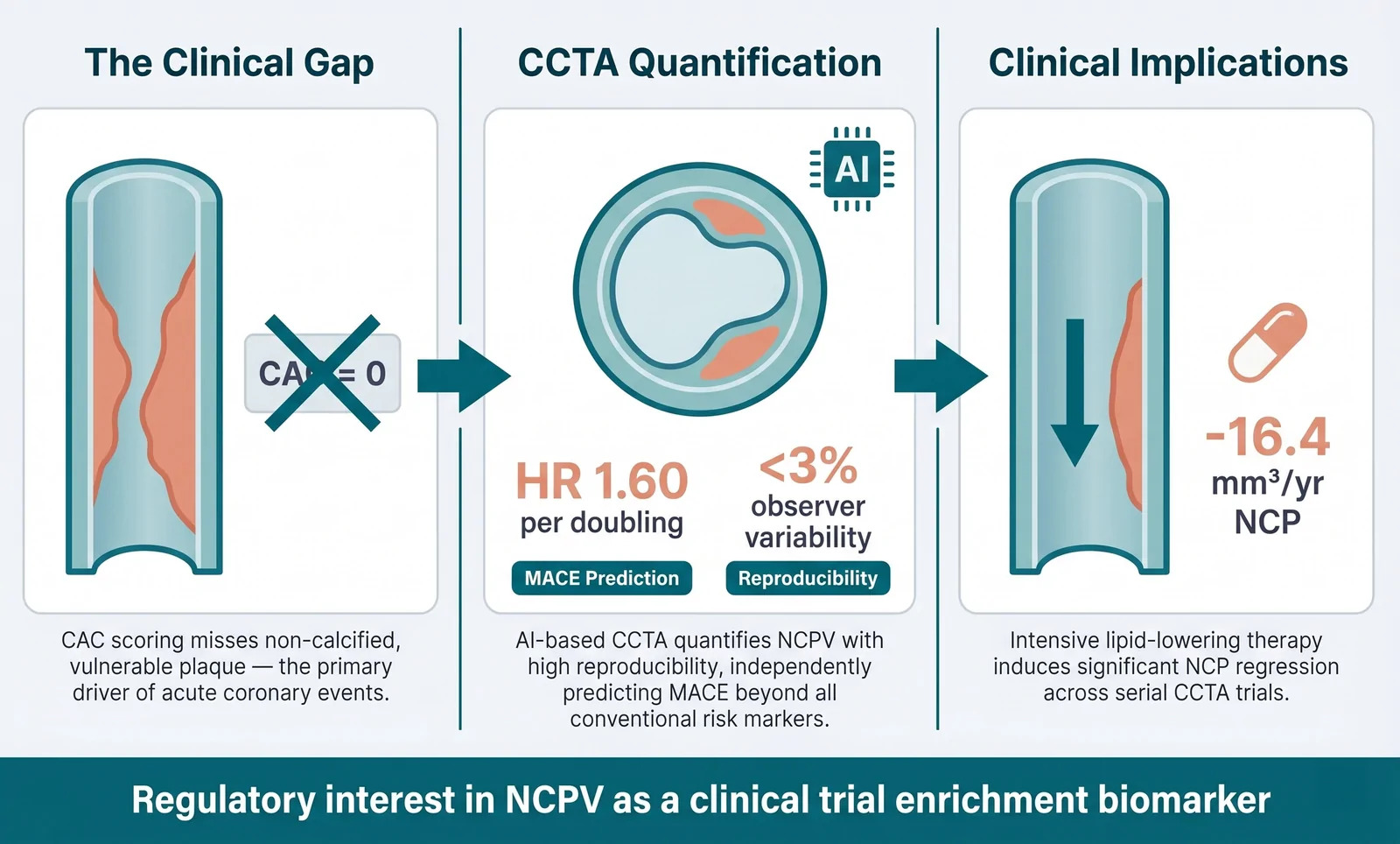
Non-Calcified Plaque Volume Assessed by Coronary Computed Tomographic Angiography: Prognostic Significance, Reproducibility, and Pharmacological Modification. Non-calcified plaque volume (NCPV) measured by coronary computed tomographic angiography (CCTA) is an emerging imaging biomarker supported by three key domains: prognostic significance, reproducibility, and responsiveness to therapy. NCPV and low-attenuation plaque burden provide incremental risk prediction for myocardial infarction beyond coronary artery calcium and stenosis severity. Artificial intelligence-based quantitative CCTA enables highly reproducible plaque measurement with low observer variability. Serial imaging studies demonstrate that NCPV is modifiable with lipid-lowering therapy. Growing regulatory interest supports its potential role as a biomarker for clinical trial enrichment. CCTA, coronary computed tomographic angiography; HR, hazard ratio; LAP, low-attenuation plaque; MI, myocardial infarction; NCP, non-calcified plaque; NCPV, non-calcified plaque volume; AI-QCT, artificial intelligence quantitative computed tomography; CAC, coronary artery calcium.

## Introduction

Coronary artery disease (CAD) remains a leading cause of morbidity and mortality worldwide, representing a persistent global public health challenge despite advances in cardiovascular prevention and therapy. An estimated 800 million individuals are affected globally, and in the USA alone, CAD accounts for approximately 610 000 deaths each year, making it the single largest contributor to mortality.^1,2^

CAD frequently progresses silently, often remaining asymptomatic until the sudden onset of an acute myocardial infarction. Plaque instability and rupture may occur long before any clinical manifestations arise, earning CAD the designation of a ‘*silent killer*’.^3^ This unpredictability underscores an urgent need for early detection strategies that can identify high-risk individuals before irreversible life-threatening events occur.

Coronary artery calcium (CAC) scoring is a well-established tool for risk stratification, with higher scores consistently associated with increased cardiovascular event rates across genders and ethnic groups.^4^ However, coronary calcification represents a relatively late stage of the atherosclerotic process, reflecting plaque stabilization rather than active vulnerability. In contrast, non-calcified plaque develops earlier, often encompassing lipid-rich, inflammation-prone components susceptible to rupture and thrombosis.^5^ Critically, the majority of myocardial infarction precursors arise from nonobstructive plaques that are invisible to CAC scoring alone, highlighting the limitations of relying solely on calcium burden for cardiovascular risk assessment.^6^

Recent advances in coronary computed tomographic angiography (CCTA) have expanded the ability to visualize and quantify atherosclerotic plaque beyond CAC scoring. Through quantitative plaque analysis, CCTA can identify and measure non-calcified plaque volume (NCPV), including low-attenuation, lipid-rich lesions that precede calcification and are prone to rupture. We conducted a narrative review of published literature through March 2025 using PubMed, synthesizing current evidence on NCPV by CCTA, focusing on its prognostic significance, reproducibility, sex- and age-related variation, and emerging role as a clinical trial enrichment biomarker.

### Search strategy and evidence selection

This narrative review was informed by a structured search of PubMed/MEDLINE for English-language articles published from database inception through March 2025. Search terms combined controlled vocabulary (MeSH) and free-text keywords using Boolean operators, including (‘coronary artery disease’ OR ‘coronary atherosclerosis’) AND (‘computed tomographic angiography’ OR ‘CCTA’ OR ‘coronary CT angiography’) AND (‘non-calcified plaque’ OR ‘low-attenuation plaque’ OR ‘plaque volume’ OR ‘quantitative plaque analysis’) AND (‘prognosis’ OR ‘major adverse cardiovascular events’ OR ‘reproducibility’ OR ‘plaque progression’ OR ‘lipid-lowering therapy’). Reference lists of key articles and relevant society consensus documents were manually screened to identify additional sources.

Priority was given to prospective multicentre trials, large registry analyses, and validation studies reporting quantitative non-calcified plaque endpoints, as well as society consensus statements and guideline documents. Studies were selected on the basis of methodological relevance, sample size, and contribution to the prognostic, reproducibility, or therapeutic-response evidence base; small single-centre series and case reports were generally excluded except where they uniquely illustrated an emerging concept. Given the narrative design, no formal quantitative pooling or risk-of-bias scoring was performed. Throughout this review, the level of supporting evidence is identified where relevant, distinguishing randomized controlled trials and prospective cohort studies from post-hoc analyses, registry data, and observational reports, so that the strength and limitations of the underlying data can be appropriately weighed. Please see *Table 1* for summary of key studies evaluating non-calcified plaque volume bt CCTA.

**Table 1:** Summary of key studies evaluating non-calcified plaque volume by CCTA

Study	Design	Population	Key NCPV finding	Evidence level
SCOT-HEART (Williams 2020)	Post-hoc analysis of RCT	1769 stable chest pain	LAP burden among strongest independent predictors of MI (HR 1.60 per doubling)	Post-hoc RCT
ICONIC (Ferraro)	Nested case–control	234 ACS cases vs. controls	Nonobstructive high-risk plaque confers risk comparable to obstructive lesions	Observational
CONFIRM (registry)	Prospective multicentre registry	>25 000 enrolled	NCP/LAP burden associated with MACE and mortality; modest incremental value in asymptomatic	Registry
PARADIGM (Lee 2018)	Prospective serial CCTA registry	1255 with serial imaging	Statins slow NCP progression and promote calcification	Registry
EVAPORATE (Budoff 2020)	Randomized placebo-controlled trial	80 with serial CCTA	Icosapent ethyl reduces LAP volume	RCT
GLAGOV (Nicholls 2016)	Randomized trial (IVUS)	968 statin-treated	PCSK9 inhibition regresses plaque by IVUS; proof-of-concept for lipid-lowering plaque regression	RCT
INVICTUS/DECODE (Rinehart 2024)	Validation studies	Multicentre	AI-QCT validated against IVUS (INVICTUS); AI quantification reclassifies management (DECODE)	Validation

ACS, acute coronary syndrome; AI-QCT, artificial intelligence quantitative computed tomography; CCTA, coronary computed tomographic angiography; HR, hazard ratio; IVUS, intravascular ultrasound; LAP, low-attenuation plaque; MACE, major adverse cardiovascular events; MI, myocardial infarction; NCP, non-calcified plaque; RCT, randomized controlled trial.

## Quantitative assessment of non-calcified plaque by CCTA

### Plaque classification and HU thresholds

CCTA provides high-resolution, three-dimensional visualization of both the coronary lumen and vessel wall, enabling direct non-invasive assessment of atherosclerotic plaque morphology and composition. Plaques are classified using Hounsfield unit (HU)-based attenuation ranges, although exact thresholds may vary across software platforms. Commonly used definitions include calcified plaque as attenuation greater than 350 HU, non-calcified plaque as intermediate attenuation tissue, and low-attenuation plaque (LAP) as less than 30 HU.^7,8^ LAP corresponds histologically to lipid-rich necrotic core and is most strongly associated with plaque vulnerability. Additional high-risk qualitative features identifiable on CCTA include positive arterial remodelling, spotty calcification, and the napkin ring sign. The napkin ring sign is defined as a low-attenuation core surrounded by a higher-attenuation rim.^9^

**Figure 1:**
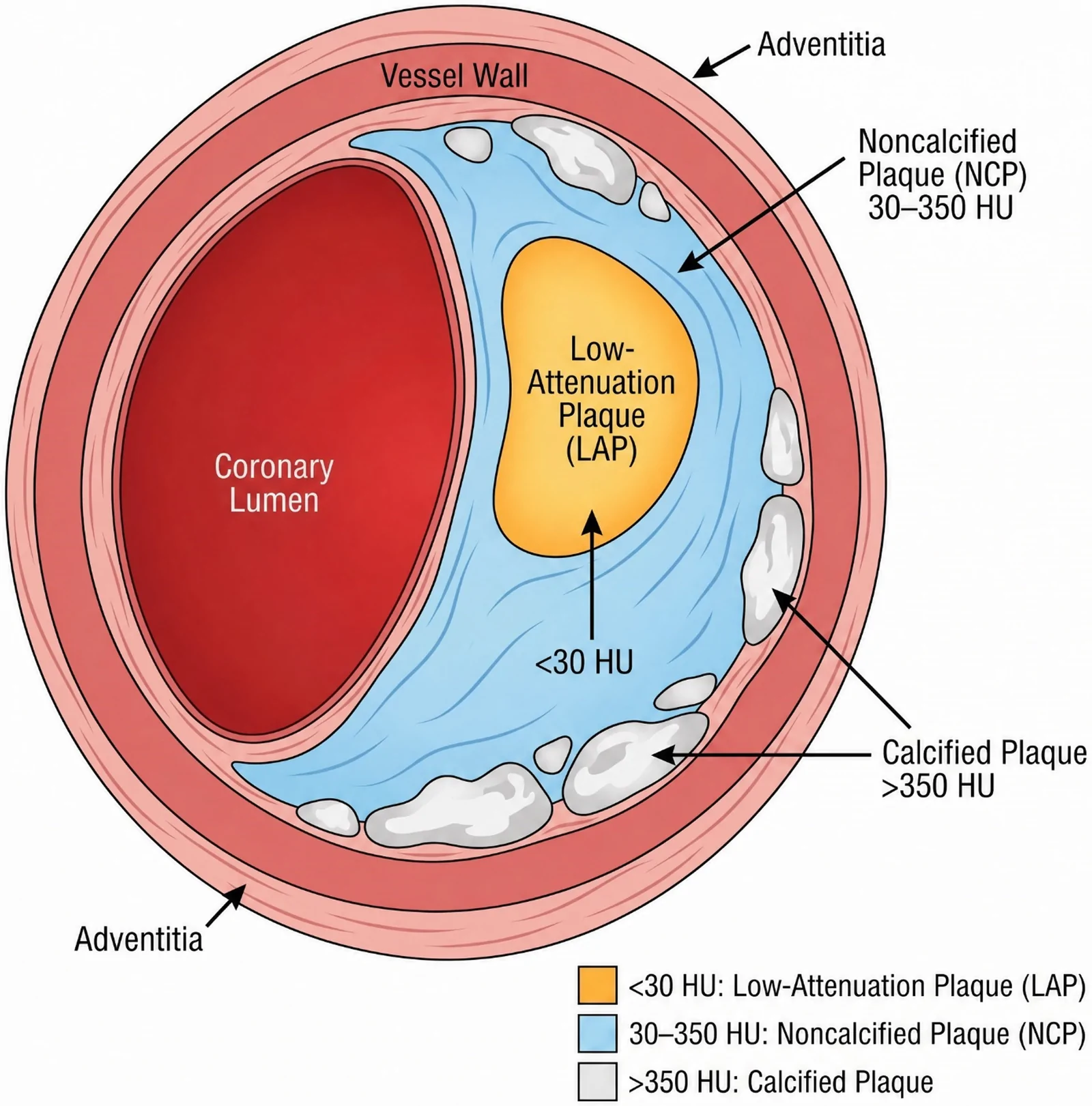
Cross-sectional illustration of coronary atherosclerotic plaque as assessed by coronary computed tomographic angiography (CCTA). Low-attenuation plaque (LAP, <30 HU; orange) represents lipid-rich necrotic core. Non-calcified plaque (NCP, 30–350 HU; blue) surrounds the LAP zone. Calcified plaque (>350 HU; grey) appears as dense nodules at the plaque periphery. HU, Hounsfield units.

Total plaque volume (TPV) represents the aggregate of all plaque subtypes, while NCPV specifically reflects the non-calcified component most susceptible to pharmacological modification and most predictive of acute coronary syndromes. *Figure 1* illustrates the cross-sectional plaque composition of a coronary artery as assessed by CCTA, with colour-coded zones representing each plaque subtype by HU threshold.

### Technical acquisition considerations

The diagnostic performance of CCTA depends on scanner design, software, and imaging protocols. Modern CCTA requires at least a 64-detector row system; newer platforms with 128–320 detector rows, dual-source, and dual-energy configurations provide superior temporal and spatial resolution with thinner collimation (0.5–0.6 mm), critical for accurate vessel wall visualization.^10^ Consistent imaging protocols and heart rate control improve image quality, and multiphasic contrast injection achieves uniform coronary opacification.^11^

An important technical consideration is the effect of tube voltage and contrast concentration on measured plaque attenuation. Data from the PARADIGM registry showed that lower tube voltage protocols increase luminal attenuation, systematically altering NCP thresholds when fixed HU values are applied.^12^ The Society of Cardiovascular Computed Tomography (SCCT) expert consensus accordingly recommends maintaining consistent luminal attenuation across serial studies and advocates for scan-specific threshold adjustment in quantitative plaque analysis protocols.

### AI-based plaque quantification platforms

Historically, quantitative coronary plaque analysis relied on manual or semi-automated segmentation, which is time-consuming and user-dependent. AI-based quantitative CT (AI-QCT) platforms, including Cleerly (Cleerly Inc., New York) and HeartFlow (HeartFlow Inc., Redwood City), have substantially streamlined plaque analysis workflows, enabling rapid, reproducible, and standardized measurements of total plaque, NCP, LAP, and calcified plaque across the entire coronary tree.^13,14^

In the INVICTUS registry, AI-QCT demonstrated high correlations with blinded IVUS core laboratory quantification of mean plaque burden and percent atheroma volume along co-registered vascular segments, supporting its use as a non-invasive surrogate for invasive plaque assessment.^15^ The DECODE study demonstrated that AI-based quantitative coronary plaque analysis prompted management reclassification in 66% of 100 clinical cases reviewed by experienced cardiologists, and 50% reclassification even when physicians reviewed CCTA images directly, suggesting that quantitative plaque data adds meaningful clinical information beyond visual interpretation alone.^16^

## Prognostic significance of non-calcified plaque volume

### NCP volume and prediction of MACE: landmark trials

**Figure 2:**
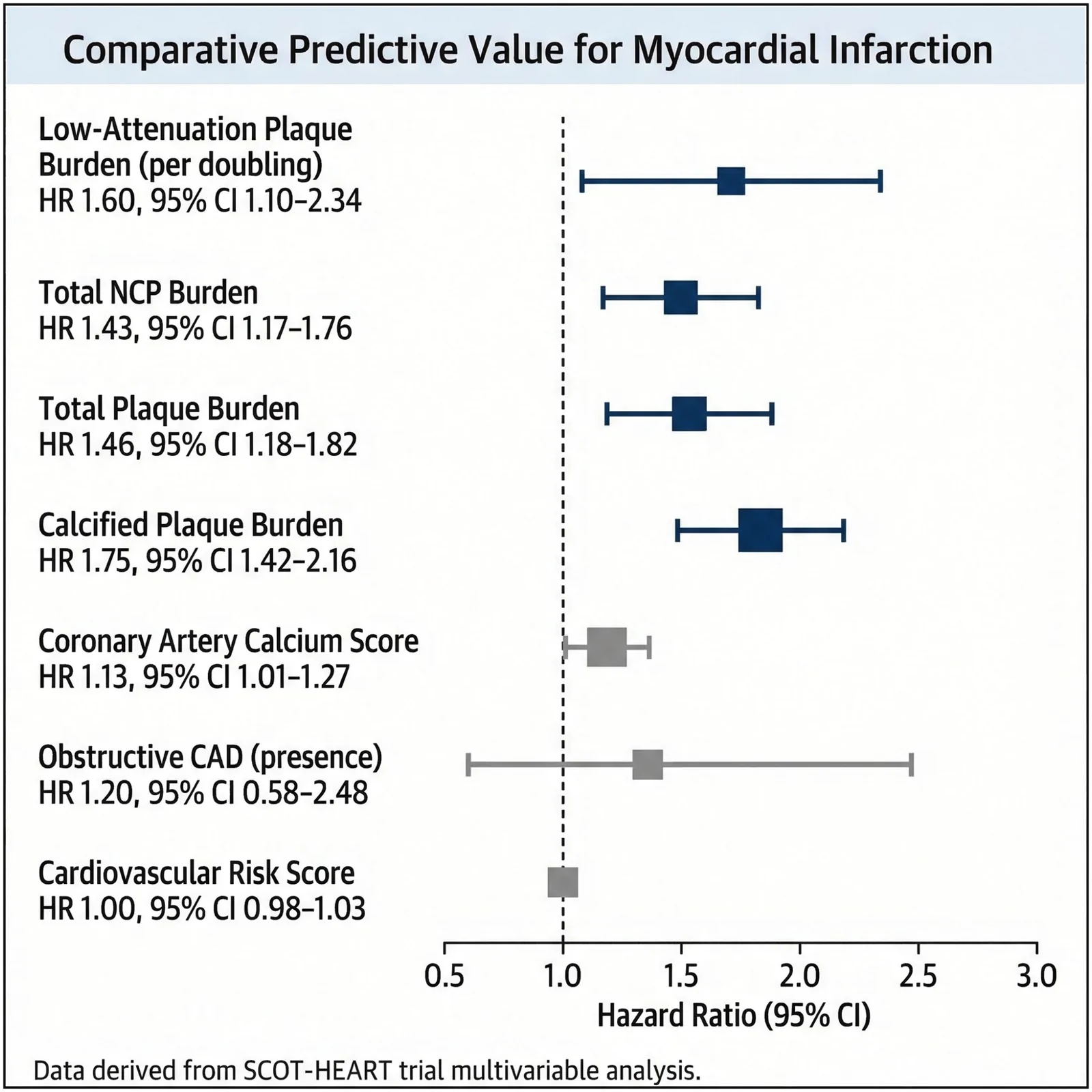
Forest plot showing comparative predictive value for myocardial infarction across plaque-based and conventional risk markers in multivariable analysis. Plaque-based predictors (navy) demonstrate substantially greater hazard ratios than conventional markers (grey). Data derived from SCOT-HEART trial multivariable analysis (Williams et al., Circulation 2020). CI, confidence interval; HR, hazard ratio; NCP, non-calcified plaque; CAD, coronary artery disease.

The most compelling evidence for the prognostic utility of NCPV derives from the SCOT-HEART trial. In a post-hoc analysis of 1769 patients with stable chest pain followed for a median of 4.7 years, Williams and colleagues demonstrated that low-attenuation NCP burden was a strong independent predictor of fatal or nonfatal myocardial infarction, outperforming cardiovascular risk scores, CAC score, and stenosis severity.^17^ LAP burden carried a hazard ratio of 1.60 per doubling (95% CI 1.10–2.34) for subsequent MI and added incrementally to all conventional risk markers. Patients with an LAP burden greater than 4% were nearly five times more likely to suffer a fatal or nonfatal MI. *Figure 2* summarizes the comparative predictive value of these markers.

Complementary data from the CONFIRM registry demonstrated that in 1226 asymptomatic individuals followed for a mean of 5.9 years, the presence of NCP or mixed plaque in one or more segments significantly increased the risk of all-cause death compared to individuals without plaque, even after adjustment for traditional risk factors. However, CCTA did not add incremental prognostic value beyond a combined model of traditional risk factors and CAC score in this asymptomatic population, suggesting that the incremental benefit of NCPV assessment may be most pronounced in symptomatic patients and those with intermediate-to-high risk.^18^

In symptomatic outpatients without known CAD analysed from the PROMISE trial, quantitative plaque measures including NCP volume and NCP burden were independently predictive of MACE after adjustment for age, sex, and cardiovascular risk factors. Optimal cut-offs of total plaque volume ≥87 mm^3^, total plaque burden ≥35%, and NCP burden ≥20% were each associated with nearly a two-fold increase in MACE risk.^19^ AI-guided quantitative plaque staging in a 536-patient cohort followed over a median of 10.3 years demonstrated incremental discriminatory value over clinical risk factors, CAC score, and conventional CCTA assessment for long-term MACE.^20^

### Nonobstructive high-risk plaques and the ICONIC study

A fundamental insight from the ICONIC (Incident Coronary Syndromes Identified by Computed Tomography) study is that nonobstructive high-risk plaques constitute the majority of culprit lesion precursors in patients who subsequently develop acute coronary syndrome. In the ICONIC nested case-control study of 234 patients with downstream ACS within the CONFIRM registry, 81.4% of high-risk plaques (HRP) were located in nonobstructive lesions (diameter stenosis <50%).^21^ Although HRP was less prevalent in nonobstructive than obstructive lesions (19.8% vs. 46.8%, *P* < 0.001), nonobstructive HRP+ lesions conferred an adjusted hazard ratio of 1.85 (95% CI: 1.26–2.72) for becoming a culprit lesion—comparable to the risk conferred by obstructive HRP-negative lesions (HR 1.41, 95% CI: 0.61–3.25). These findings demonstrate that HRP should be identified and reported even in the setting of nonobstructive CAD, and highlight the critical importance of full plaque characterization beyond luminal stenosis severity.

Within the ICONIC study, plaque location and vessel geometry also emerged as independent determinants of culprit lesion risk. Lesions located at vessel bifurcations, tortuous segments, or at shorter distances from the coronary ostium were more likely to develop into culprit lesions independent of plaque morphology and quantitative plaque characteristics.^22^ An increasing number of adverse geometric characteristics (AGCs) was associated with up to a seven-fold greater risk of future culprit lesions, with additive discriminatory value beyond plaque burden and high-risk morphological features.

### Sex- and age-related differences in NCP volume

Sex- and age-related differences in coronary plaque composition have important implications for CCTA-based risk stratification. In a sub-analysis of the ICONIC study patients prior to ACS, Conte and colleagues demonstrated that older patients (age ≥65 years) had higher total plaque volume than younger patients, driven primarily by greater calcified plaque volume (135.9 ± 163.7 vs. 63.8 ± 94.2 mm^3^, *P* < 0.0001). In contrast, females had significantly lower fibrous and fibrofatty plaque volumes than males (fibrofatty volume: 29.6 ± 44.1 vs. 75.3 ± 98.6 mm^3^, *P* = 0.0001), while no sex-related differences were found in the prevalence of qualitative high-risk plaque features.^23^ These findings underscore that women tend to express atherosclerosis with less calcified and less fibrofatty NCP burden, with important implications: sex should be considered during CCTA-based plaque quantification, and risk stratification approaches relying on calcified plaque measures alone may systematically underestimate risk in women.

### NCP volume vs. CAC scoring

The relationship between NCPV and CAC score is complex. While moderately correlated (*r* = 0.62 in SCOT-HEART), they capture distinct biological processes: CAC reflects stabilized disease, while NCP, particularly LAP, represents biologically active vulnerable atheroma.^17^ In multivariable analyses including both NCPV and CAC score, the incremental prognostic value of CAC is substantially attenuated, whereas NCPV retains independent significance for MACE prediction.^17^ CAC = 0 patients may harbour non-trivial NCP burden, particularly younger individuals and women. AI-based plaque quantification from the CONFIRM II registry demonstrated that increasing NCP and LAP burden were associated with elevated MACE risk in women, reinforcing the need for direct NCP quantification.^24^

## Reproducibility of non-calcified plaque volume measurement

### Inter- and intra-observer variability

Robust reproducibility is a fundamental prerequisite for the clinical and research utility of NCPV. Early studies using 64-detector row CCTA established excellent intra-observer and interobserver reproducibility: in a seminal study by Hoffmann and colleagues evaluating 30 patients who underwent dual CCTA acquisitions within 200 days, intra-observer-interscan correlations for total, calcified, and NCP volumes were excellent (*r* = 0.93–0.97, *P* < 0.001).^25^ Notably, these serial reproducibility data derive from relatively small cohorts, and further validation in larger, multicentre populations is necessary to fully establish the reliability of serial NCPV quantification for longitudinal monitoring.

**Figure 3:**
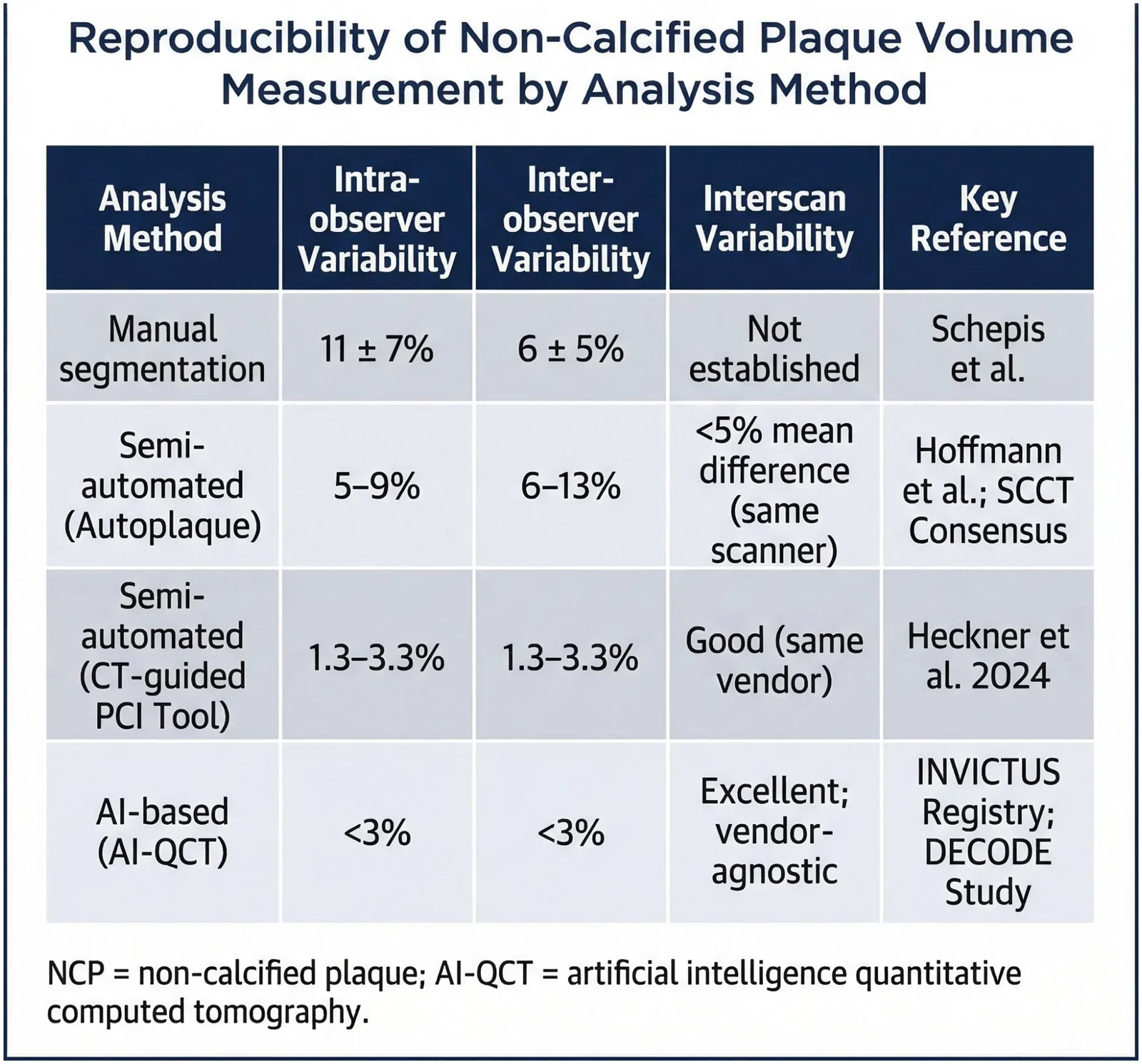
Summary table of reproducibility metrics for non-calcified plaque volume measurement by analysis method. Automated AI-based platforms demonstrate the lowest inter- and intra-observer variability with vendor-agnostic interscan reproducibility. NCP, non-calcified plaque; AI-QCT, artificial intelligence quantitative computed tomography.

Subsequent studies confirmed that interobserver variability for NCP volume measurement ranges from approximately 6% to 13% with semi-automated platforms, and 1.3–3.3% with more highly automated approaches.^26^ Schepis and colleagues specifically evaluated NCP volume reproducibility in 70 patients, reporting a mean interobserver difference of 6 ± 5% and an intra-observer variation of 11 ± 7%, with CT mean plaque volume closely matching IVUS measurements (89 ± 66 mm^3^ vs. 90 ± 73 mm^3^).^27^  *Figure 3* provides a consolidated comparison of reproducibility metrics across analysis methods.

A key distinction exists between the reproducibility of different plaque subtypes. Calcified plaque demonstrates the highest reproducibility due to high HU contrast, while LAP shows the greatest variability due to low HU values and proximity to the vessel wall, where partial volume effects can substantially affect segmentation boundaries.^28^ Total NCP volume, integrating all non-calcified components, is more reproducible than LAP alone and is therefore the preferred primary endpoint in serial pharmacological trials.

### Interscan and platform reproducibility

The SCCT Expert Consensus on Standards for Quantitative CCTA Assessment reports that mean differences for total plaque volume across serial scans with semi-automated tools are typically below 5% when performed on the same scanner. However, substantial variance has been reported for LAP and calcified plaque, and reproducibility decreases across different CT platforms.^12^ Based on observed interscan reproducibility, an interventional trial using NCP as a primary CCTA endpoint would require approximately threefold larger sample sizes compared to IVUS-based trials.

In patients with advanced coronary artery disease, despite imaging quality challenges from calcification-related artefacts, intra-observer, interobserver, and interscan correlations remained excellent for total plaque and NCP volumes at the per-patient level.^29^ Current generation semi-automated software yields intraclass correlation coefficients of 0.91–0.92 for TPV and 0.87–0.91 for NCPV with intercepts of 15–22 mm^3^ and slopes of 0.98–0.99, confirming high reliability for serial assessment.^29^

### FDA recognition and ARC consensus

The reproducibility and prognostic importance of NCPV have attracted formal regulatory attention. The CAD Frontiers ACTION A2D2 Consortium, a multidisciplinary group including representation from the US Food and Drug Administration (FDA), has identified CCTA-derived NCPV as a priority candidate for clinical trial enrichment. A Biomarker Qualification Program Letter of Intent has been submitted to the FDA, reflecting growing interest in NCPV as a potential biomarker for clinical trial enrichment.^30^ The Consortium concluded that CCTA-derived NCPV measures are reproducible, closely correlate with IVUS measures, and have strong prognostic ability for MACE—meeting the FDA's definition of a prognostic biomarker used to identify the likelihood of clinical events in patients with the disease of interest.

## Temporal progression and pharmacological modification of NCP volume

### Natural history of NCP progression

Serial CCTA studies have provided important insights into the natural history of NCP over time. In a prospective long-term study of 202 patients with a mean interscan period of 6.2 years, both total and non-calcified plaque volumes increased progressively. Hypertension was the primary modifiable risk factor associated with NCP progression, whereas diabetes mellitus was predominantly associated with calcified plaque progression.^31^ These observations suggest that different cardiovascular risk factors may have differential effects on distinct plaque components, with implications for tailored risk management.

The PARADIGM (Progression of AtheRosclerotic PlAque Determined by Computed TomoGraphic Angiography IMaging) registry enrolled patients with suspected or known CAD undergoing serial CCTA at least 2 years apart, providing seminal data on plaque progression rates and the differential effects of therapy.^32^ This registry established serial CCTA as a feasible and informative tool for atherosclerosis monitoring, and demonstrated that quantitative plaque measures can detect biologically meaningful changes in NCP volume over clinically relevant follow-up intervals.

### Effect of statin therapy on NCP volume

**Figure 4:**
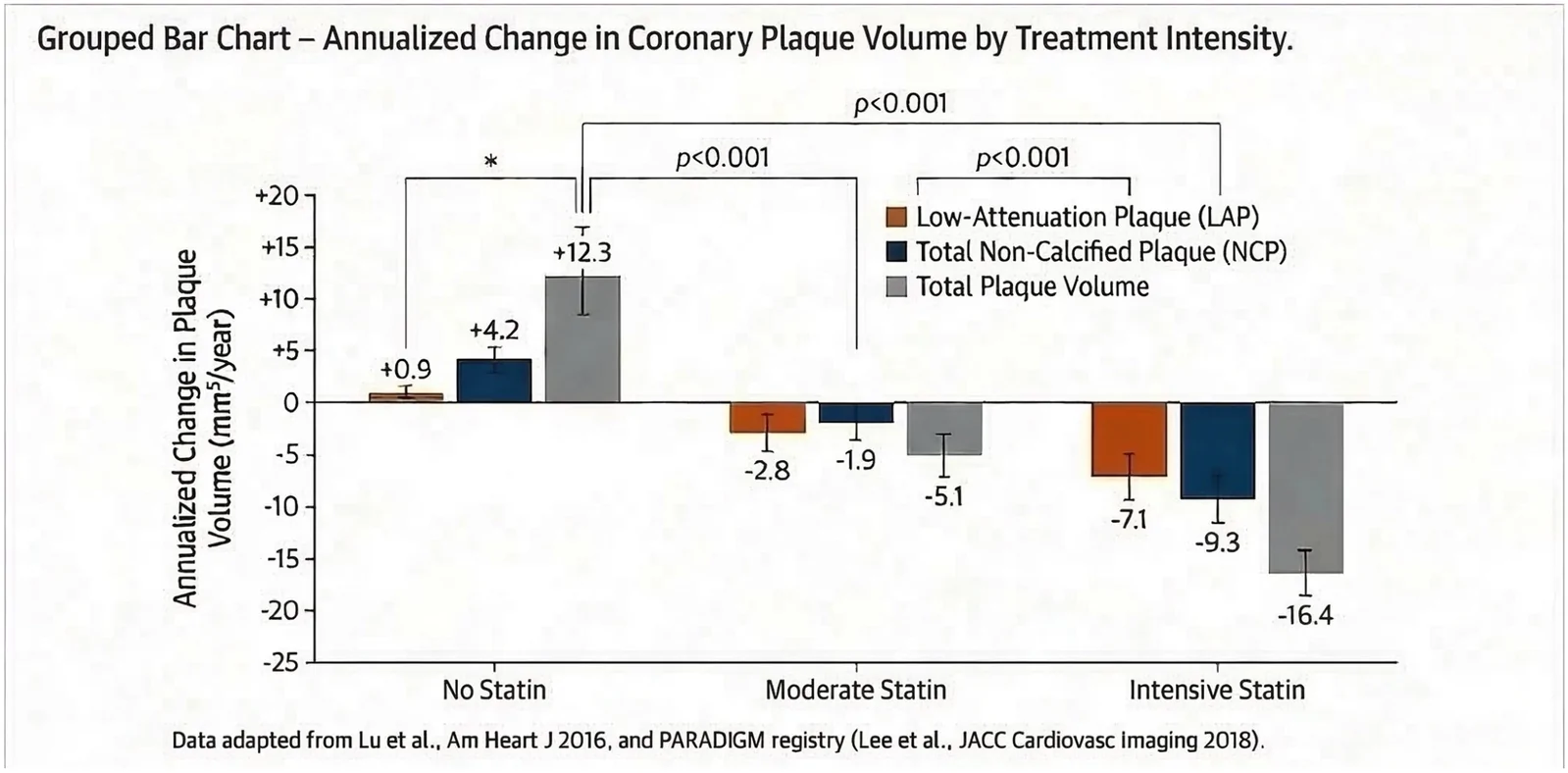
Annualized change in coronary plaque volume by treatment intensity, showing low-attenuation plaque (LAP), total non-calcified plaque (NCP), and total plaque volume across no statin, moderate statin, and intensive statin groups. Intensive statin therapy is associated with significant regression of all NCP subtypes. Data adapted from Lu et al., Am Heart J 2016, and the PARADIGM registry (Lee et al., JACC Cardiovasc Imaging 2018).

Statin therapy is the most extensively studied pharmacological intervention for its effects on coronary plaque composition as assessed by CCTA. Multiple serial CCTA studies demonstrate that statin treatment—particularly intensive therapy—is associated with significantly attenuated NCP progression and, in some studies, regression of NCP volume. *Figure 4* summarizes annualized changes in plaque volume components across treatment intensity groups.

In a multicentre prospective study of 206 patients with mild NCP on baseline CCTA, intensive statin therapy was associated with significant regression of LAP volume (annualized change: −7.1 ± 13.1 vs. +0.9 ± 12.7 mm^3^, *P* < 0.001), total plaque volume (−16.4 ± 35.0 vs. +12.3 ± 32.4 mm^3^, *P* < 0.001), and percent plaque volume compared to no statin treatment. Patients with greater baseline LAP volume showed the greatest benefit from statin therapy.^33^

The PARADIGM study further demonstrated that statin-treated patients showed reduced plaque volume progression, greater plaque calcification, and a lower likelihood of developing high-risk plaque features.^32^ Statin therapy was associated with larger decreases in LAP and fibro-fatty plaque volumes and larger increases in dense calcified plaque—consistent with pharmacological plaque stabilization through conversion of vulnerable NCP to more calcified, stable forms. A JACC State-of-the-Art Review confirmed that statins consistently reduce NCP, fibro-fatty, and necrotic core volumes, while fibrous and calcified volumes increase—a pattern reflecting stabilization rather than regression.^34^

### Effect of additional lipid-lowering therapies

The EVAPORATE trial evaluated 4 grams per day of icosapentaenoic acid (IPE) vs. placebo on coronary plaque progression using serial CCTA in 80 patients over 18 months, and demonstrated significant regression of LAP volume and attenuation of overall NCP progression with IPE—providing a biological correlate for the MACE reduction observed in the REDUCE-IT cardiovascular outcomes trial.^35^ The GLAGOV trial established proof-of-concept for NCP regression with intensive LDL-C lowering via PCSK9 inhibition (evolocumab) using serial IVUS, and subsequent CCTA studies have confirmed that reductions in LDL-C are associated with decreases in NCP volume while calcified plaque increases.^36^

### NCPV as a clinical trial endpoint

The SCCT Expert Consensus identifies NCP volume as among the most clinically meaningful parameters for assessing therapeutic response, citing its demonstrated sensitivity to pharmacological intervention and its association with downstream cardiovascular events.^12^ Key considerations for CCTA-based NCP endpoints in clinical trials include consistent acquisition protocols across study visits, standardized analysis software and attenuation thresholds, and use of the same CT platform throughout the study period. Updated CAD-RADS 2.0 reporting guidelines now incorporate overall plaque burden estimation as a required element of CCTA reporting.^37^ The FDA's acceptance of a Biomarker Qualification Program Letter of Intent for NCPV as a trial enrichment biomarker in December 2024 further solidifies its standing as an emerging regulatory-grade endpoint in cardiovascular drug development.^30^

## Clinical applications and future directions

### Integration into risk stratification pathways

**Figure 5:**
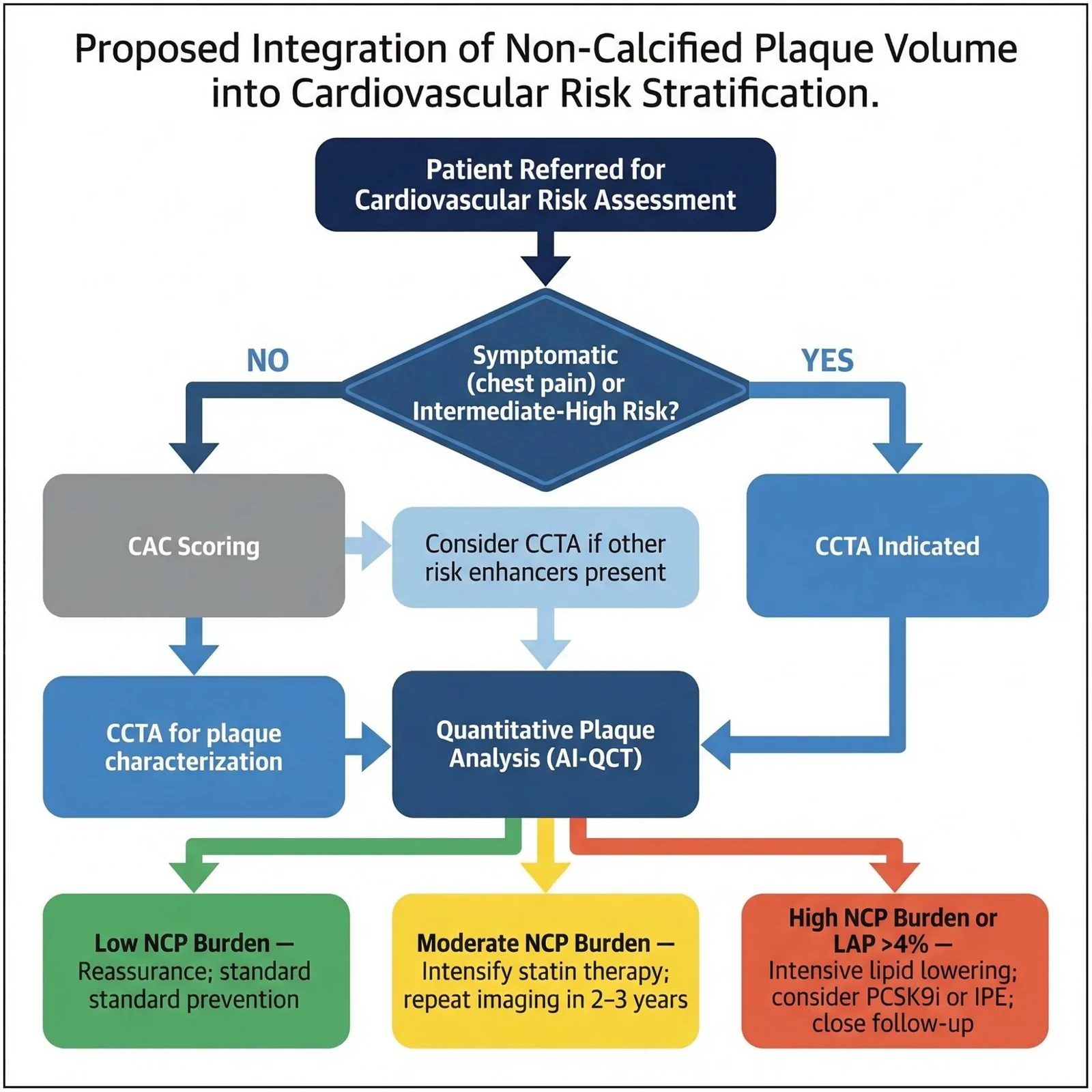
Proposed clinical decision pathway for the integration of non-calcified plaque volume (NCPV) into cardiovascular risk stratification. Patients are triaged by symptom status and risk level towards CAC scoring or CCTA, with quantitative AI-based plaque analysis guiding therapeutic intensity based on NCP burden. CAC, coronary artery calcium; CCTA, coronary computed tomographic angiography; AI-QCT, artificial intelligence quantitative computed tomography; LAP, low-attenuation plaque; NCP, non-calcified plaque; PCSK9i, proprotein convertase subtilisin/kexin type 9 inhibitor; IPE, icosapentaenoic acid ethyl ester.

The accumulating evidence supports integration of quantitative NCPV assessment into clinical cardiovascular risk stratification pathways. Identification of high NCP burden—even in the absence of obstructive stenosis or elevated CAC scores—may trigger intensification of preventive therapies, more aggressive LDL-C targets, and closer clinical follow-up. AI-based plaque staging systems that classify patients into discrete stages based on percent atheroma volume have demonstrated 10-year prognostic value with practical clinical applicability.^20^  *Figure 5* presents a proposed clinical decision pathway integrating NCPV into cardiovascular risk stratification.

### Special populations

Several populations may derive particular benefit from NCPV-based risk stratification. Patients with CAC=0 represent a clinically important subgroup in whom NCP burden may be elevated despite absent calcification, particularly younger individuals and women. Among stroke patients without known cardiac disease, CCTA-based plaque characterization demonstrated prognostic utility, with NCP and high-risk plaque types associated with significantly higher MACE rates than calcified plaque alone.^38^ Women, whose atherosclerosis tends to manifest with less calcified and less fibrofatty plaque for equivalent risk factor burden, may require direct NCP quantification rather than reliance on CAC alone to avoid systematic underestimation of risk—a finding directly supported by ICONIC sub-study data.^23,24^

### Limitations and future directions

Despite significant progress, several limitations persist. First, spatial resolution constraints of current CT systems limit detection of thin fibrous caps, precluding reliable identification of thin-cap fibroatheroma on CCTA. Second, significant inter-scanner and inter-vendor variability in HU thresholds necessitates careful standardization across sites, and reproducibility decreases when different CT platforms are used for serial assessments.^12^ Third, while NCP burden is well-established as prognostically significant in symptomatic chest pain populations, data in asymptomatic primary prevention cohorts remain more limited, with the CONFIRM registry suggesting modest incremental value over combined traditional risk factors and CAC in asymptomatic individuals.^18^ Fourth, practical barriers to widespread clinical adoption warrant consideration. Quantitative NCP analysis requires dedicated software, computational infrastructure, and reader expertise that add cost and time relative to standard CCTA interpretation, and access to AI-based quantification platforms remains uneven across health systems. The incremental cost-effectiveness of NCPV-guided management over established risk-assessment pathways has not yet been established in prospective studies. Furthermore, the threshold at which NCPV findings should alter clinical decision-making—such as initiation or intensification of preventive therapy—remains undefined, and some analyses have reported attenuation of the independent prognostic value of plaque volume after adjustment for comprehensive clinical risk factors and CAC, underscoring that the incremental yield of NCPV may vary across populations and clinical contexts.

Future directions include the development of vendor-neutral AI platforms that enable accurate NCP quantification across diverse CT systems, prospective evaluation of NCPV-guided therapeutic intensification in randomized trials, and integration of NCPV with complementary CT-derived biomarkers such as pericoronary adipose tissue (PCAT) attenuation- a marker of coronary inflammation that has demonstrated additive predictive value for MI beyond LAP burden in SCOT-HEART.^39^ The emergence of photon-counting CT technology will require development of new acquisition and analysis protocols optimized for NCPV quantification, offering the potential for further improvements in plaque characterization accuracy and reproducibility.

## Conclusion

Non-calcified plaque volume assessed by CCTA has emerged as a reproducible and prognostically relevant measure of coronary atherosclerosis. Evidence from large multicentre studies demonstrates that NCPV provides incremental information beyond CAC and stenosis severity, particularly in identifying vulnerable, non-obstructive plaque. Quantitative plaque analysis, supported by advances in artificial intelligence and standardized imaging protocols, offers a promising approach for refining cardiovascular risk assessment and monitoring therapeutic response. Continued efforts towards standardization, validation, and integration into clinical workflows will be essential to define the role of NCPV in routine practice and clinical trial design.

## Data Availability

No new data were generated or analysed for this review. All data discussed are available in the cited published literature.
